# Importance of Echocardiography and Clinical “Red Flags” in Guiding Genetic Screening for Fabry Disease

**DOI:** 10.3389/fcvm.2022.838200

**Published:** 2022-04-25

**Authors:** Rodolfo Citro, Costantina Prota, Donatella Ferraioli, Giuseppe Iuliano, Michele Bellino, Ilaria Radano, Angelo Silverio, Serena Migliarino, Maria Vincenza Polito, Artemisia Ruggiero, Rosa Napoletano, Vincenzo Bellizzi, Michele Ciccarelli, Gennaro Galasso, Carmine Vecchione

**Affiliations:** ^1^Heart Department, University Hospital San Giovanni di Dio e Ruggi d'Aragona, Salerno, Italy; ^2^Vascular Physiopathology Unit, Department of Angio-Cardio-Neurology, IRCCS Neuromed, Pozzilli, Italy

**Keywords:** Fabry disease, myocardial hypertrophy, echocardiography, screening, genetic

## Abstract

**Introduction:**

Aim of this study was to evaluate, in a metropolitan area not already explored, the prevalence of Anderson–Fabry disease, by genetic screening, in patients with echocardiographic evidence of left ventricular hypertrophy (LVH) of unknown origin and “clinical red flags”.

**Methods:**

From August 2016 to October 2017, all consecutive patients referring to our echo-lab for daily hospital practices with echocardiographic evidence of LVH of unknown origin in association with history of at least one of the classical signs and symptoms related to Fabry disease (FD) (neuropathic pain, anhidrosis/hypohidrosis, angiokeratomas, gastrointestinal problems, chronic kidney disease, or cerebrovascular complications) were considered eligible for the FD genetic screening program. Through dried blood spot testing, α-Galactosidase A (α-Gal A) activity and analysis of the *GLA* gene were performed.

**Results:**

Among 3,360 patients who underwent transthoracic echocardiography in our echo-lab during the study period, 30 patients (0.89%; 19 men, mean age 58 ± 18.2 years) were selected. FD was diagnosed in 3 (10%) unrelated patients. Three different *GLA* gene mutations were detected, one of them [mutation c.388A > G (p.Lys130Glu) in exon 3] never described before. Moreover, probands' familiar genetic screening allowed the identification of 5 other subjects affected by FD.

**Conclusion:**

In a metropolitan area not previously investigated, among patients with LVH of unknown origin associated with other “red flags,” undergoing genetic screening, the prevalence of FD was very high (10%). Our results highlight the importance of an echocardiographic- and clinical-oriented genetic screening for FD in patients with uncommon cause of LVH.

## Introduction

Left ventricular hypertrophy (LVH) is a common finding associated to a large variety of conditions, such as essential hypertension, aortic stenosis, hypertrophic cardiomyopathy, and more rarely inherited metabolic disorders including Fabry disease (FD) (1).

Fabry disease is a X-linked lysosomal storage disorder caused by enzyme α-Galactosidase A (α-Gal A) deficit due to *GLA* gene mutations, leading to progressive tissue accumulation of glycosphingolipids, especially globotriaosylceramide (Gb3) and globotriaosylsphingosine (lyso-Gb3). Despite being considered rare with an original incidence ranging from 1:40,000 to 1:117,000 live births, screening studies in newborns and in high-risk populations have found higher frequencies ranging from 1:1,600 to 1:8,000 (2–5).

Clinical manifestations are usually slowly progressive, with a variable time of onset, severity, and course, making challenging the correct identification of FD and the differential diagnosis with other pathologies. The most frequent signs or symptoms are angiokeratomas, lenticular and corneal opacity, microalbuminuria, or proteinuria until chronic kidney disease and peripheral and central nervous system disorders (acroparesthesia, anhidrosis/hypohidrosis, and stroke). With regard to cardiac involvement, Gb3 accumulation in myocytes leads to LVH resulting usually in concentric remodeling. Furthermore, the involvement of conduction tissue and valve leaflets often induce arrhythmias and valvular heart diseases (6).

Several studies have already investigated the prevalence of FD in adult subjects with unexplained LVH. However, frequency of patients with FD in such a highly selected populations has been variable, from 0 to 12%, reflecting referral and gender bias and differences in diagnostic methodology (Table 1) (7–25).

**Table 1:** Previous screening studies of FD in patients with LVH.

**Screening study 7–25)**	**Population**	**Screening selection criteria**	**Screening methods**	**Prevalence of FD**	**Limitations**
Nakao et al. (7)	230 males	LVH≥13 mm	α-Gal A activity	3%	Only males Tertiary referral center In 5/7 cases, no mutation was found in *GLA* gene
Sachdev (8)	153 males	HCM≥13 mm	α-Gal A activity + genetic	3.9%	Only males Tertiary referral center Exclusion hypertension and valvular disease
Ommen et al. (9)	100 (44 males)	Obstructive HCM	Myomectomy: electron microscopy	0	Asymmetric hypertrophy is rare in FD Tertiary referral center Exclusion hypertension and valvular disease
Chimenti (10)	34 females	HCM	Endomyocardial biopsy: electron microscopy + α-Gal A activity + genetic	12%	Biopsy study Tertiary referral center
Arad et al. (11)	75 (45 males)	HCM≥13 mm	Genetic	0	Tertiary referral center Exclusion hypertension and valvular disease
Morita et al. (12)	1,862, 50 LVH (41 males)	LVH≥13 mm	Genetic	2%	Exclusion hypertension and valvular disease
Monserrat et al. (13)	508 (328 males)	HCM	α-Gal A activity (only if low, genetic)	1%	Use of α-Gal A activity in females Exclusion hypertension and valvular disease
Havndrup et al. (14)	90 (56 males)	HCM	Genetic	3%	Tertiary referral center
Hagège et al. (15)	392 (278 males)	HCM≥15	α-Gal A activity	1.5%	Use of α-Gal A activity in females Exclusion hypertension and valvular disease High cut-off for LVH
Elliot et al. (16)	1,386 (885males)	HCM≥15	Genetic	0.5%	Exclusion hypertension and valvular disease High cut-off for LVH
Terryn et al. (17)	540 (362 males)	LVH≥13 mm	α-Gal A activity + genetic	0.9%	/
Mawatari et al. (18)	738 males	LVH≥13 mm	α-Gal A activity (only if low, genetic)	0	Only males
Palecek et al. (19)	100 males	LVH≥13 mm	α-Gal A activity (only if low, genetic)	4%	Only males
Gaggl et al. (20)	2,593 (1,686 males)	LVH≥12 mm	Urinary Gb3 concentration (only if high, α-Gal A activity + genetic)	0	Low sensitivity of screening method (many false negatives)
Kubo et al. (21)	177 males	HCM≥15	α-Gal A activity + genetic + lyso-Gb3	1.1%	Only males High cut-off for LVH
Cecchi et al. (22)	235	HCM	Myomectomy: electron Microscopy + genetic	1.3%	Low sensitivity of screening methods (only 3 pts were analyzed for the visual suspect of storage disease by surgeon)
Maron et al. (23)	585, 413 males	HCM	α-Gal A activity + genetic	0.34%	Tertiary referral center
Barman et al. (24)	80	Non-obstructive HCM	α-Gal A activity + genetic	2.5%	Only males Exclusion hypertension and valvular disease
Kim et al. (25)	988 males	LVH≥13 mm	α-Gal A activity (only if low, genetic)	0.3%	Only males Exclusion valvular disease

*LVH, left ventricular hypertrophy; FD, Fabry disease; α GAL-A, α-Galactosidase A enzyme; HCM, hypertrophic cardiomyopathy*.

The aim of this study was to evaluate, in a metropolitan area not already explored, the prevalence of Anderson–Fabry disease, by genetic screening, in patients with echocardiographic evidence of LVH of unknown origin and “clinical red flags.”

## Methods

### Study Population and Data Collection

We prospectively enrolled for FD genetic screening men and women of 18 years or older with echocardiographic evidence of LVH of unknown origin and at least one “red flag.”

Patients with maximal wall thickness in end-diastole ≥13 mm, independently of LVH pattern (concentric and asymmetric) among all consecutive patients referring to our echo-lab for daily hospital practices and cardiologic examination from August 2016 to October 2017, were selected (26).

In this subgroup, the search for “red flags” considered classical signs and symptoms related to FD [neuropathic pain, anhidrosis/hypohidrosis, angiokeratomas, gastrointestinal problems (nausea, vomiting, abdominal pain, diarrhea, or constipation), chronic kidney disease, or cerebrovascular complications (transient ischemic attack or stroke)] was performed (27).

The only exclusion criterion was represented by LVH of already known etiology, such as genetic hypertrophic cardiomyopathy or other inherited metabolic disorders. Patients with arterial hypertension, defined as ≥140 mmHg systolic blood pressure or being on antihypertensive medication (28), and severe aortic stenosis, assessed through an echocardiographic step-wise integrated approach (29), were included.

All variables of patients selected for genetic screening were recorded on a standardized form that including information on patient demographics (sex, age, heart rate, systolic, and diastolic blood pressure), medical history, signs and symptoms at presentation, family history for cardiovascular, cerebrovascular, or renal diseases, electrocardiographic features, laboratory exams, and echocardiographic parameters.

All patients enrolled gave written informed consent, and the study was approved by the local ethics committee.

### Echocardiographic Measurements

A commercially available cardiac ultrasonography system (GE Vivid E80, General Electric, Milwaukee, Wisconsin) with a 2.5–4.5-MHz phased-array transducer with second harmonic capability was used for complete 2-dimensional Doppler echocardiography. All examinations were performed by cardiologists and those of the patients selected for genetic screening reviewed by one expert reader (RC). Measurements were performed according to the European Guidelines of Chamber Quantification. Particularly, septal or posterior wall thickness was measured by 2D-guided M-mode at papillary muscle level in parasternal long axis or parasternal short axis view at end-diastole (at onset of R-wave); LV ejection fraction (EF) was calculated using biplane Simpson's rule from the apical four- and two-chamber views (26). LV diastolic function was evaluated according to the American Society of Echocardiography recommendations, including mitral flow velocities, mitral annular e′ velocity, and E/e′ ratio (30). Mitral regurgitation (MR) was also quantified from color Doppler imaging and semiquantitatively graded as absent, mild, moderate, or severe, using standardized criteria (29). Degree of severity of aortic stenosis was assessed through an echocardiographic step-wise integrated approach as recommended by current guidelines (29). In addition, global longitudinal strain (GLS) was assessed by using automated speckle-tracking echocardiography (STE) in four- and two-chamber and apical long axis views, respectively (30).

### Dried Blood Spot Testing and Genetic Analysis

Peripheral blood of patients included in the genetic screening program was collected by medical residents, using ethylenediaminetetraacetic acid (EDTA) as an anticoagulant. Patient's blood was embedded on dedicated filter paper obtaining dried blood spots (DBSs), which were sent to Centogene Laboratories (Rostock, Germany). Both enzyme activity determination and genetic screening were performed by staff blinded to clinical data. In male patients, α-Gal A activity was measured as first; in case of absent or low α-Gal A (n.v. ≥ 15.3 μmol/l/h), diagnosis was confirmed by mutation analysis of the *GLA* gene. In female patients, genetic mutation analysis was the primary screening tool. Plasma concentration of lyso-Gb3, a degradation product of the accumulating Gb3, was also evaluated (n.v. ≤ 1.8 ng/ml) (31). All genetic variants were classified according to American College of Medical Genetics (ACMG) recommendations.

Patients with a first positive test for FD underwent a second peripheral blood collection in order to confirm the diagnosis.

## Results

### Clinical Features of the Study Population

Over a study period of 14 months, a total of 3,360 patients underwent transthoracic echocardiography in our echo-lab; 30 patients (0.89%; 19 men, 63.3%) with a mean age of 58 ± 18.2 years, due to the finding of suspected hypertrophy and at least one “red flag,” were considered eligible for the genetic screening program.

In overall genetically screened population, 17 (56.6%) patients had a systolic blood pressure of ≥140 mmHg or were on hypertensive medication; 9 (30%) patients had chronic kidney disease or were in dialysis; only 2 (6.7%) patients had an history of cerebrovascular disease with previous stroke; about half of the studied population (16, 53.3%) reported personal or familiar history of cardiovascular disorders (acute myocardial infarction, arrhythmias, and sudden cardiac death). Among other typical but not pathognomonic FD manifestations, about one-third patients (10, 33.3%) were concerned about unspecific gastrointestinal problems since childhood, whereas only two (6.7%) manifested both acroparesthesia and hypohidrosis. Nobody exhibited typical angiokeratomas.

In the 30 patients who underwent genetic screening, sinus rhythm on ECG was documented in most patients (24, 80%), whereas atrial fibrillation and pacemaker rhythm were detected in 5 (16.7%) and 1 (3.3%) patients, respectively. The mean heart rate was 71 ± 15 beats per minute (bpm) (range 45–110), with a PR interval of 169 ± 34 ms (range 108–250) and a prevalence of atrioventricular and intraventricular conduction disorders (both right and left bundle branch blocks) of 8.5 and 23.4%, respectively.

Left ventricular hypertrophy was detected in all patients who underwent genetic screening, with a mean value of the interventricular septum thickness in tele-diastole of 15.4 ± 3.6 mm (range 13–22) and posterior ventricular wall of 16.2 ± 2.1 mm (range 13–23). About a quarter of patients (24.8%) showed signs of type I diastolic dysfunction (E/A = 0.75 ± 0.11; E/è = 13 ± 4.9). Moderate-to-severe MR was detected in 4 patients (13.3%), whereas only one had a severe aortic stenosis, with a mean transvalvular gradient of 45 mmHg. Echocardiographic parameters of patients who undergone genetic screening are shown in Table 2.

**Table 2:** Main echocardiographic findings of FD patients.

**Index case**	**Max left ventricular thickness (mm)**	**Left ventricular end-diastolic volume (ml/mq)**	**Left ventricular end-systolic volume (ml/mq)**	**Ejection fraction (%)**	**Left atrial volume (ml/mq)**	**Diastolic dysfunction grade**	**Valvular disease**	**TAPSE (mm)**	**Left ventricular global longitudinal strain (%)**
1	14	54	24	56	28	I (impaired relaxation phase)	None	25	−18.7
2	17	75	39	48	44	I (impaired relaxation phase)	Mild mitral regurgitation	21	−11.6
3	17	74	33	55	47	I (impaired relaxation phase)	Mild-moderate mitral regurgitation	22	−8.9
4 (Patient #1 son)	12	72	22	69	40	I (impaired relaxation phase)	Mild mitral regurgitation	27	−18.8
5 (Patient #2 daughter)	0,9	48	19	60	24	Normal diastolic function	None	25	−20.6
6 (Patient #2 grandchild)	0,6	44	13	70	21	Normal diastolic function	None	27	−22
7 (Patient #3 brother)	17	91	58	36	49	II (pseudonormal phase)	Moderate mitral regurgitation; moderate tricuspidal regurgitation	17	−6.3
8 (Patient #3 brother)	16	74	31	58	42	II (pseudonormal phase)	Mild mitral regurgitation	21	−15

### Genetic Screening

Three different *GLA* gene mutations (10% of screened population) in three unrelated patients were detected. One of them [mutation c.388A > G (p.Lys130Glu) in exon 3] was a novel variant just described from our group (32), whereas the other two [mutation c.901C > G (p.Arg301Gly) in exon 6; mutation c.337T > C (p.Phe113Leu) in exon 2] have been previously reported (33, 34). Probands' familial screening allowed the identification of five other FD patients.

### Fabry Disease Patients

Baseline characteristics of FD patients detected by our genetic screening program and their relatives were showed in Table 3.

**Table 3:** Clinical and molecular data of the three FD cases and their relatives.

**Index case**	**Gender/age**	**Signs and symptoms**	**α GAL-A Activity (μmol/l/h)**	***GLA* Mutation**	**LYSO-GB3 Levels (ng/ml)**
1	F/57	Acroparesthesias, headache, abdominal pain	Normal	c.388A>G (p.Lys130Glu), exon 3	4
2	M/66	Mild chronic kidney disease	<0.8	c.901C>G (p.Arg301Gly), exon 6	5.3
3	M/69	Paroxysmal atrial fibrillation, gastrointestinal problems, arterial hypertension	<2.8	c.337T>C (p.Phe113Leu), exon 2	15.3
4 (Patient #1 son)	M/25	Acroparesthesias, abdominal pain	<0.8	c.388A>G (p.Lys130Glu), exon 3	18.1
5 (Patient #2 daughter)	F/41	None	Normal	c.901C>G (p.Arg301Gly), exon 6	Normal
6 (Patient #2 grandchild)	M/11	Developmental disability, history of epilepsy, gastrointestinal problems	<0.8	c.901C>G (p.Arg301Gly), exon 6	3.9
7 (Patient #3 brother)	M/76	CAD,PMK implantation for AV block, mild chronic kidney disease	<0.8	c.337T>C (p.Phe113Leu), exon 2	12.5
8 (Patient #3 brother)	M/74	Arterial hypertension	<0.8	c.337T>C (p.Phe113Leu), exon 2	9.5

*CAD, coronary artery disease; PMK, pacemaker; AV block, atrio-ventricular block*.

Patient #1 is a 57-year-old woman with a history of acroparesthesias, recurrent headache, and abdominal pain, who was admitted to our echo-lab because of a cardiovascular screening program dedicated to hospital employees, in the absence of known cardiac problems and/or family history. ECG showed a sinus rhythm with a cardiac frequency of 60 bpm without abnormalities of repolarization. Echocardiography showed concentric LVH with a septum of 14 mm and a normal systolic left ventricular ejection fraction (LVEF = 56%, but a type I diastolic dysfunction. GLS was within lower range (−18.7%; n.v. from −15.9 to −22.1%). Neurological examination revealed no apparent deficit, as ophthalmologic examination (performed after the FD diagnosis) did not show signs of tortuous vessels or cornea verticillata. Her laboratory exams also resulted in the normal range, particularly no signs of chronic kidney disease were detected. α-Gal A activity was normal (≥15.3 μmol/l/h), but genetic examination showed a heterozygous mutation in exon 3 of the *GLA* gene, c.388A > G (p.Lys130Glu), a novel variant just described from our group. Plasma concentration of lyso-Gb3 (4.0 ng/ml, n.v. ≤ 1.8 ng/ml) was pathologically elevated. On these grounds, this novel mutation was considered as pathogenic. Furthermore, among the patient's relatives, the same gene mutation was observed in her son, who also showed decreased α-Gal A enzymatic activity (<0.8 μmol/l/h; n.v. ≥ 15.3 μmol/l/h) and elevated lyso-Gb3 levels (18.1 ng/ml; n.v. ≤ 1.8 ng/ml), leading to the diagnosis of overt FD.

Patient #2 is a 66-year-old man with mild chronic kidney disease who was admitted to our hospital for cardiac arrest. His past medical history and family history were both negative for cardiovascular risk factors and diseases. ECG showed signs of inferior myocardial infarction so urgent coronary angiography and subsequent percutaneous coronary intervention with drug-eluting stent on right coronary artery was performed. Echocardiographic examination demonstrated a severe LVH with a septum of 17 mm and a mild systolic left ventricular dysfunction with an LVEF of 45%. In addition, also GLS was reduced (−11.6%). Biochemical and genetic tests confirmed both low α-Gal A activity (<0.8 μmol/l/h; n.v. ≥ 15.3 μmol/l/h) and the *GLA* gene mutation in exon 6 c.901C > G (p.Arg301Gly). Elevated lyso-Gb3 levels were also collected (5.3 ng/ml; n.v. ≤ 1.8 ng/ml). Family screening revealed the same *GLA* gene mutation in his 41-year-old daughter, without clinical manifestations of the disease, and in her 11-year-old son, who presented with developmental disability.

**Figure 1:**
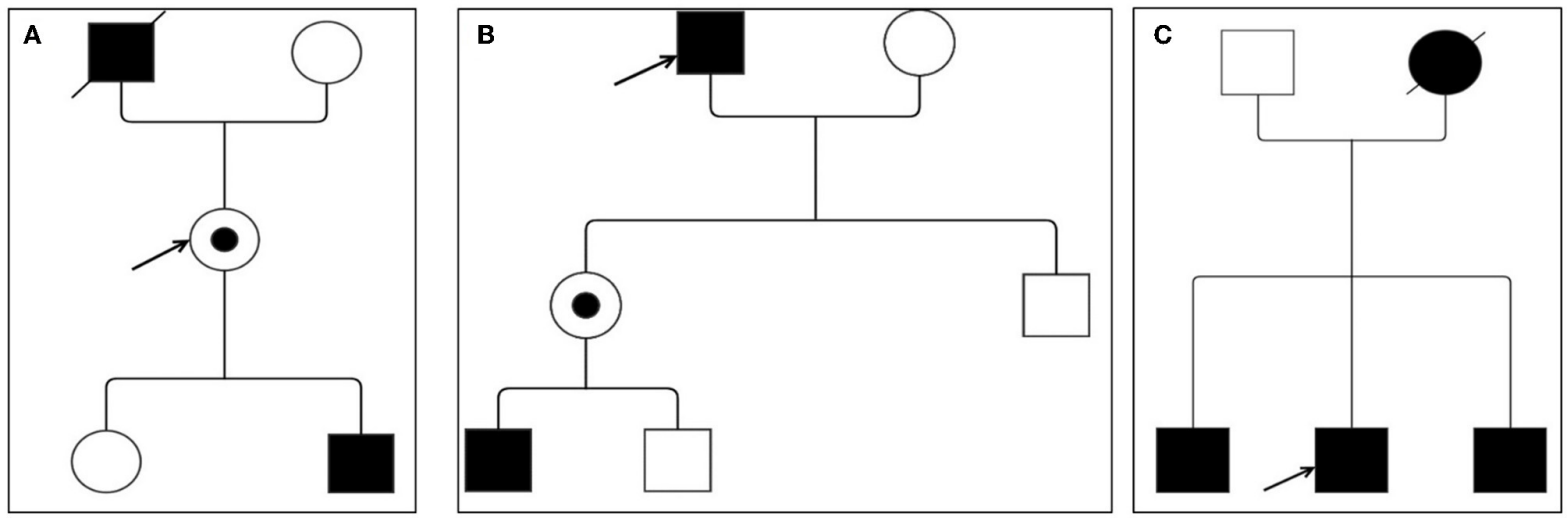
Family pedigree of Fabry disease (FD) patients. **(A)** Patient #1; **(B)** patient #2; and **(C)** patient #3. Circles are women and squares are men. Filled symbols are affected patients. Dashed symbols are deceased patients. The arrow indicates the index patient. Dotted means carriers without symptoms.

Patient #3 is a 69-year-old man referring to our laboratory for a cardiologic follow-up due to a recent diagnosis of arterial hypertension. Past medical history revealed a previous episode of paroxysmal atrial fibrillation and a history of gastrointestinal problems during young age. Moreover, his mother had unspecified cardiac problems. ECG showed a sinus rhythm with a cardiac frequency of 77 bpm and criteria for LVH, whereas clinical examination resulted unremarkable; in addition, there was no evidence of chronic kidney disease at laboratory exams. Echocardiography demonstrated a severe concentric LVH with a septum of 17 mm and a preserved systolic LVEF = 55%, but a highly reduced GLS value of −8.9%. Biochemical and genetic tests confirmed both low α-Gal A activity (<2.8 μmol/l/h; n.v. ≥ 15.3 μmol/l/h) and the *GLA* gene mutation c.337T > C (p.Phe113Leu) in exon 2. Plasma concentration of lyso-Gb3 (15.3 ng/ml, n.v. ≤ 1.8 ng/ml) was highly elevated. Furthermore, among the patient's relatives, the same gene mutation was detected in his two brothers: one had a past medical history of coronary artery disease and bypass graft, pacemaker implantation for a third-degree atrioventricular block at 67 years old, and mild chronic kidney disease; the second one was a patient with hypertension with no other remarkable medical history; both patients showed a severe concentric LVH at echocardiographic examination (Figure 1).

## Discussion

Our study highlights, in daily clinical practice, the importance of FD genetic screening in the evaluation of patients with echocardiographic evidence of myocardial hypertrophy of unknown origin and at least one “red flag.” The main findings can be summarized as follows:

- High FD prevalence of 10% among patients with LVH associated with other “red flags” in a metropolitan area not previously investigated was found;
- Hypertension and aortic stenosis should not be considered exclusion criteria for FD screening in suspicious patients with LVH;
- A new mutation [c.388A > G (p.Lys130Glu) in exon 3 of the *GLA* gene] causing a classical phenothype of FD has been reported (32).

Cardiovascular involvement in FD substantially occurs due to disease-related morbidity and mortality of these patients (7); correct diagnosis in a very early stage and specific treatment are fundamental to modify the natural course of the disease (35).

**Figure 2:**
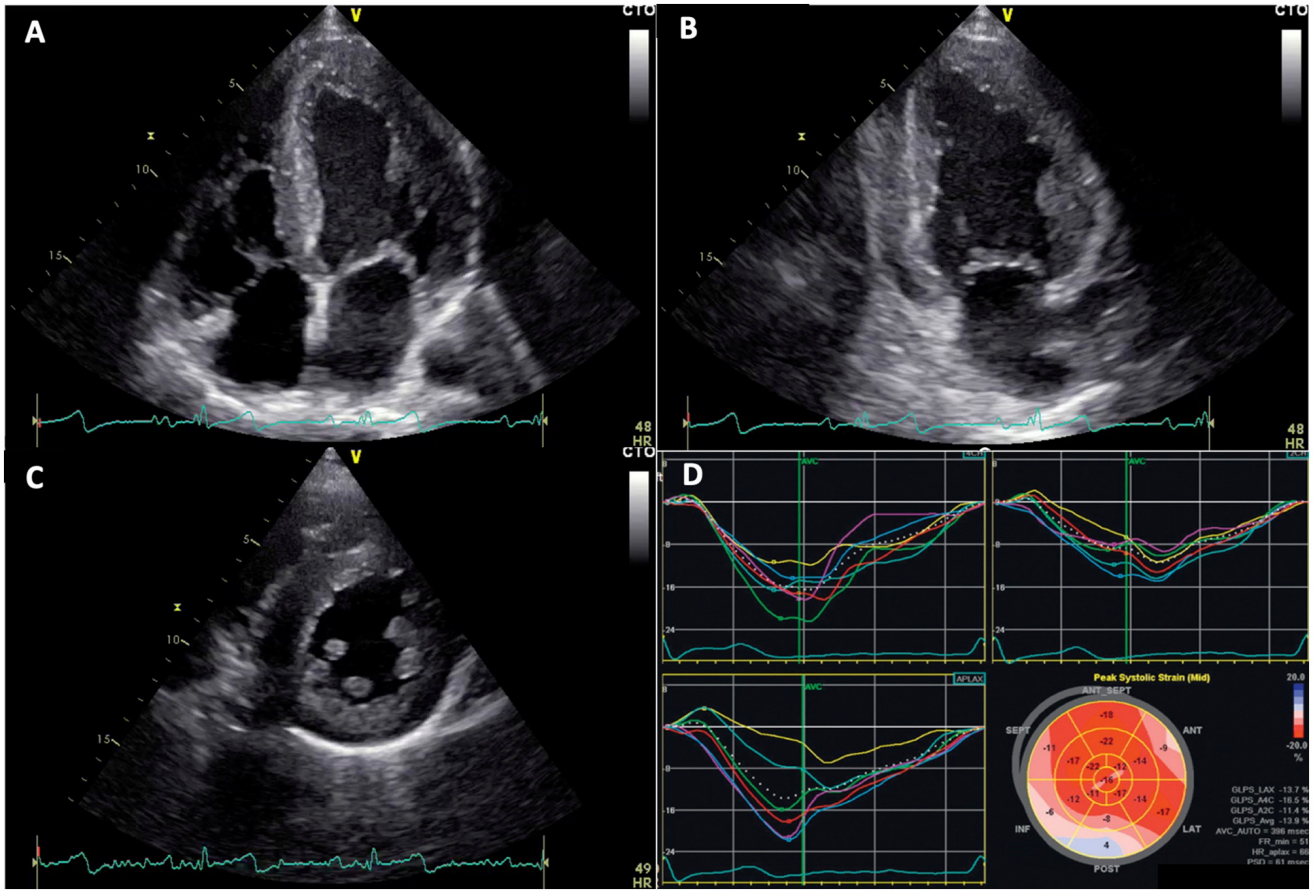
Echocardiographic images of Fabry disease (FD) patients. **(A–C)** apical 4 chambers, 2 chambers, and parasternal short axis views showing left ventricular concentric hypertrophy typical of FD, respectively. **(D)** altered global longitudinal strain in patient with FD, note inferolateral segment alterations.

Usually, accumulation of glycosphingolipids in myocardial cells leads to ventricular remodeling and subsequent concentric hypertrophy, even if asymmetric hypertrophy has also been rarely described (9, 21). Echocardiography represents the primary diagnostic tool in detection of LVH (1) (Figure 2). However, the proportion of patients with FD who are not correctly recognized at the time of echocardiography or first medical contact is up to date still high. Despite the availability of fast and easy to perform genetic screening techniques to determine the enzymatic activity of α-Gal A or genetic mutations in the *GLA* gene, the real incidence of FD is probably still underestimated (6).

Interestingly, our results reveal a higher prevalence (10%) of FD among patients with LVH of unknown origin associated with at least one sign or symptom suspect for FD compared to previous screening studies (7–25). They are consistent with Chimenti (2004), which reported a similar prevalence of 12% in female patients with hypertrophic cardiomyopathy (10).

A possible explanation of this finding is attributable to an important selection bias among different studies since the screened population has been selected with different inclusion criteria. In general, hypertension on its own is responsible for LVH (28). Hence, patients with hypertension are almost systematically excluded from screening for metabolic heart diseases. We decided to include patients with hypertension with suspect of FD because the two disorders can coexist: hypertension could be both an unrelated comorbidity and conversely a consequence of FD, since LysoGb3 accumulation causes intima media thickening and vascular pathology (36). The exclusion of patients with hypertension from our screening program would have let to miss a potential diagnosis of FD.

Similarly, severe aortic stenosis is usually an exclusion criterion in similar screening investigations since pressure overload could explain myocardial hypertrophy. Considering aortic stenosis, the most common primary valve disease in Europe, with a growing prevalence due to the aging population (29), we decide to also include patients with known history of severe aortic stenosis if clinical suspicion of FD raised. The only subject with severe aortic stenosis included in our screening has been resulted, but not affected by FD. Anyway, the key message is that besides the presence of a possible explanation for myocardial hypertrophy, suspicious signs and symptoms for FD should alert cardiologists to investigate deeply even mild and non-specific manifestations, to avoid missing the FD diagnosis.

Nowadays, more than 940 mutations (Human Gene Mutation Database, http://www.hgmd.org, last accessed February 2022) in the coding *GLA* regions, on the long arm of chromosome X (Xq22), are known (37).

The identification of novel mutations through a well-performed screening represents an important goal nowadays, not only for increasing molecular knowledge of *GLA* gene but especially for the important clinical implications derived from the detection of asymptomatic or not very symptomatic patients with FD in a very early phase. Recognizing this mutation, in fact, allows for the prompt initiation of therapies and cascade family screening of immediate and extended family.

Utilizing DBS testing is very easy and fast to screen for FD in patients with otherwise undiagnosed LVH. Detection at an early phase is crucial, due to the availability of effective therapies most beneficial when started in the first stages of the disease's course. Indeed, a prompt instauration of enzyme replacement therapy (ERT) or other new specific treatment, before the organ involvement becomes irreversible, has the great potential to positively influence the natural history of the disease reducing morbidity and mortality (38). In our study, DBS testing and clinical assessment was able to successfully identify not only three patients with FD but also five relatives with FD in a preclinical phase, underlying the importance for physicians to be aware of this opportunity in evaluating patients with LVH.

Moreover, in our study, some patients with FD, despite normal global systolic function expressed by EF, revealed reduced or slightly reduced GLS values, confirming previous reported results (39, 40) where GLS appears to be more sensitive than LVEF to detect subclinical myocardial dysfunction in this peculiar setting.

## Limitations

A recent study showed that using plasma to measure enzymatic activity of Gal A might fail to detect some male patients with FD, affirming that the gold standard should be determination of α-Gal A activity in leukocytes (41). On the contrary, other reports demonstrated that biochemical screening through DBS is generally accepted to be very sensitive in screening in men, and DBS is probably as accurate as the gold standard (31). The study includes a small number of patients to draw definitive conclusions about the prevalence in our area.

In addition, regarding the new *GLA* gene mutation, there is a general agreement that a histologic demonstration of Gb3 storage in one of the target tissues is desirable before classifying a genetic variant of uncertain significance as pathogenic; unfortunately, we are not able to provide diagnostic confirmation in this circumstance. However, pathologically elevated plasma concentration of lyso-Gb3 in the index case, in addition to the finding of the same mutation in her son, who showed also decreased α-Gal A enzymatic activity, can be considered as strong markers of overt FD (42).

## Conclusion

Compared with previous studies performed in highly selected populations in tertiary treatment centers, our study population is more “generic” and involves patients of a metropolitan area not already explored for FD, but better representing a mirror of real world. Among patients with LVH associated with other “red flags,” undergoing genetic screening program, the prevalence of FD was very high (10%). Our results highlight the importance of an echocardiographic and clinical-oriented FD genetic screening and encourage its extensive use in patients with uncommon cause of LVH for prompt recognition of patients affected by this peculiar genetic disorder.

## Data Availability Statement

The original contributions presented in the study are included in the article/supplementary material, further inquiries can be directed to the corresponding author.

## Ethics Statement

The studies involving human participants were reviewed and approved by Ospedale San Giovanni di Dio e Ruggi d'Aragona. Written informed consent to participate in this study was provided by the participants' legal guardian/next of kin.

## Author Contributions

All authors listed have made a substantial, direct, and intellectual contribution to the work and approved it for publication.

## Conflict of Interest

The authors declare that the research was conducted in the absence of any commercial or financial relationships that could be construed as a potential conflict of interest.

## Publisher's Note

All claims expressed in this article are solely those of the authors and do not necessarily represent those of their affiliated organizations, or those of the publisher, the editors and the reviewers. Any product that may be evaluated in this article, or claim that may be made by its manufacturer, is not guaranteed or endorsed by the publisher.
